# Autotaxin Activity in Chronic Subdural Hematoma: A Prospective Clinical Study

**DOI:** 10.3390/diagnostics12081865

**Published:** 2022-08-02

**Authors:** Theodosis Kalamatianos, Evangelos Drosos, Christiana Magkrioti, Ioanna Nikitopoulou, Christos Koutsarnakis, Anastasia Kotanidou, George P. Paraskevas, Vassilis Aidinis, George Stranjalis

**Affiliations:** 1 Department of Neurosurgery, Faculty of Health Sciences, School of Medicine, Evaggelismos General Hospital, National and Kapodistrian University of Athens, 106 76 Athens, Greece; drosose@gmail.com (E.D.); ckouts@hotmail.co.uk (C.K.); gstranjalis@med.uoa.gr (G.S.); 2 Hellenic Centre for Neurosurgery Research, “Professor Petros S. Kokkalis”, 106 75 Athens, Greece; 3 Institute for Fundamental Biomedical Research, Biomedical Sciences Research Center Alexander Fleming, 166 72 Athens, Greece; magkrioti@fleming.gr (C.M.); aidinis@fleming.gr (V.A.); 4 GP Livanos and M Simou Laboratories, 1st Department of Critical Care & Pulmonary Services, School of Medicine, Evaggelismos General Hospital, National and Kapodistrian University of Athens, 106 76 Athens, Greece; joannaniki@gmail.com; 5 1st Department of Critical Care & Pulmonary Services, School of Medicine, Evaggelismos General Hospital, National and Kapodistrian University of Athens, 106 76 Athens, Greece; akotanid@med.uoa.gr; 6 2nd Department of Neurology, School of Medicine, “Attikon” General Hospital, National and Kapodistrian University of Athens, 124 62 Athens, Greece; gparask@med.uoa.gr

**Keywords:** chronic subdural hematoma, autotaxin, lysophosphatidic acid, beta trace, serum, hematoma fluid, dura

## Abstract

Autotaxin (ATX) is the ectoenzyme producing the bulk of lysophosphatidic acid (LPA) in circulation. ATX and LPA-mediated signaling (the ATX-LPA axis) play critical roles in the vascular and nervous system development. In adults, this axis contributes to diverse processes, including coagulation, inflammation, fibroproliferation and angiogenesis under physiological and/or pathophysiological conditions. Given evidence implicating several of these processes in chronic subdural hematoma (CSDH) pathogenesis and development, we assessed ATX activity in CSDH patients. Twenty-eight patients were recruited. Blood and hematoma fluid were collected. Enzymatic assays were used to establish serum and hematoma ATX activity. Enzyme-linked immunosorbent assays were used to establish hematoma beta trace (BT) levels, a cerebrospinal fluid (CSF) marker, in a hematoma. ATX activity was nearly three folds higher in hematoma compared to serum (P < 0.001). There was no significant correlation between BT levels and ATX activity in a hematoma. The present results show, for the first time, that ATX is catalytically active in the hematoma fluid of CSDH patients. Moreover, our findings of significantly elevated ATX activity in hematoma compared to serum, implicate the ATX-LPA axis in CSDH pathophysiology. The CSF origin of ATX could not be inferred with the present results. Additional research is warranted to establish the significance of the ATX-LPA axis in CSDH and its potential as a biomarker and/or therapeutic target.

## 1. Introduction

Initially discovered as a motility stimulating factor in human melanoma cells [1], autotaxin (ATX) was later purified from fetal bovine serum and recognized as the secreted enzyme catalyzing the production of the bioactive phospholipid lysophosphatidic acid (LPA) [2]. ATX and associated LPA-signaling (the ATX-LPA axis) have since been implicated in numerous mammalian physiological and pathophysiological processes. Thus, genetic deletion and overexpression experiments in mice have firmly established the critical role of ATX in normal nervous and vascular system development and in the production of the bulk of circulating LPA [3,4,5]. Moreover, LPA-signaling has been shown to drive a diverse range of cellular processes, including platelet aggregation, as well as proliferation and migration of fibroblasts and immune and endothelial cells. These actions have been implicated not only in physiological responses to tissue injury allowing wound healing, but also in the pathophysiology of chronic inflammatory/fibrotic disease including pulmonary, liver or renal fibrosis and in atherosclerosis (reviewed by [6,7]). In this context, loss of the negative feedback effect of LPA on ATX expression via induction of the ATX gene by inflammatory cytokines, allowing sustained elevation of ATX and LPA levels, has been proposed as a hallmark of chronic inflammatory disease, including cancer (reviewed by [8]). Preclinical evidence indicating the developmental roles of LPA signaling in sprouting angiogenesis suggests the utility of this system as a novel therapeutic target for pathological angiogenesis [9].

Chronic subdural hematoma (CSDH) represents one of the most common neurosurgical entities with an increasing incidence driven, at least partly, by the demographic shift towards ageing populations [10]. Our interest in the ATX-LPA axis in CSDH stems from considerable evidence indicating that several of the processes attributed to the aforementioned acute or sustained activity of this axis including coagulation, fibroproliferation, inflammation and angiogenesis have been implicated in CSDH pathogenesis, development recurrence (for a comprehensive review on CSDH pathophysiology, see [11]). Of note, previous clinical evidence indicating the presence of the cerebrospinal fluid (CSF) marker beta trace in CSDH suggests the involvement of CSF leaks and mixing of CSF with blood during CSDH pathogenesis [12]. Additional, clinical imaging evidence indicates a transition from a subdural hygroma to a CSDH in a proportion of cases [13]. In this context, it is noteworthy that ATX is synthesized in the human choroid plexus [14]. It is abundantly present and active in human CSF and at higher levels compared to circulation [15,16]. Moreover, LPA synthesis has been shown to dramatically increase in CSF following incubation with serum, the latter, unlike CSF, is a rich source of the enzyme’s substrate lysophosphatidylcholine (LPC) [15]. Thus, the pathogenesis of CSDH can be postulated to involve an initial surge of LPA synthesis. This surge may contribute to several processes, such as granulation tissue formation, exemplified by the CSDH external membrane displaying evidence for fibroproliferation and angiogenesis [11], in a manner analogous to physiological wound healing [7]. In this regard, the external membrane of CSDH, which is considered the locus driving hematoma development, contains smooth muscle cells, active fibroblasts, immature/permeable capillaries, as well as inflammatory cells [11]. Bleeding from the immature, permeable capillaries in CSDH has been postulated to be a possible mechanism for hematoma expansion [11]. Some additional clinical evidence points towards the involvement of distal branches of the middle meningeal artery in angiogenic processes of the external membrane in CSDH [17].

In the present prospective clinical study, we have begun addressing the putative involvement of the ATX-LPA axis in CSDH by examining levels of ATX activity in CSDH fluid and have investigated the hypothesis that CSF is a potential source for ATX in CSDH by incorporating analysis of the CSF marker beta trace.

## 2. Materials and Methods

### 2.1. Patient Selection and Biofluids

Patient inclusion criteria were as follows: all patients admitted to the Department of Neurosurgery, Evaggelismos Hospital, Athens, Greece, who were diagnosed with CSDH and consented to participate prior to their first-ever burr hole surgery and during a two-year period (1 January 2018–1 January 2020) were eligible. Patient exclusion criteria were: patients with acute SDH and those with renal insufficiency, liver dysfunction/disease, history of psychiatric disorder or neoplastic disease [12,18,19,20,21]. Patient demographic characteristics, medical history, neurological examination results and computerized tomography (CT) findings were recorded. Peripheral blood was obtained on the day and prior to the burr hole surgery, the latter allowing for the collection of subdural hematoma fluid samples. Blood and hematoma fluid were processed in an identical manner as described previously [22] and stored at −80 °C until assayed.

### 2.2. LysoPLD Activity Assay

ATX has Lysophospholipase D (LysoPLD) activity, catalyzing the cleavage of lysophosphatidylcholine (LPC) to LPA and choline. LysoPLD activity was measured with the TOOS activity assay in which the released choline was oxidised with choline oxidase to betaine and hydrogen peroxide. The latter reacts with the reagents N-ethyl-N-(2-hydroxy-3-sulfopropyl)-3-methylaniline (TOOS) and aminoantipyrene (4-AAP) in the presence of horseradish peroxidase (HRP) to form a pink quinoneimine dye absorbing at 555 nm. In particular, LysoPLD buffer (100 mM Tris-HCl pH 9.0, 500 mM NaCl, 5 mM MgCl_2_, 5 mM CaCl_2_, 120 μM CoCl_2_ and 1 mM LPC) was prewarmed at 37 °C for 30 min and subsequently, biological samples (4 μL of serum or hematoma) were mixed with 96 μL of the LysoPLD buffer and incubated at 37 °C for 3 h in a 96-well plate. At the end of the incubation, 100 μL of a color mix (0.5 mM 4-AAP, 7.95 U/mL HRP, 0.3 mM TOOS, 2 U/mL choline oxidase in 5 mM MgCl_2_/50 mM Tris- HCl pH 8.0) were added to each well. Absorbance (A) was measured at 555 nm every 5 min for 20 min. Absorbance was plotted against time to determine the time frame where the reaction is linear and dA/dT (sample)-dA/dT (blank) was calculated. Saline instead of the sample was used as a blank. ATX activity was calculated according to the equation: Activity (U/mL) = [dA/dT (sample)-dA/dT (blank)] × Vt/(e × Vs × 0.5), where T, time (min); Vt, total volume of reaction (ml); Vs, volume of sample (ml); e, milimolar extinction coefficient of quinoneimine dye under the assay conditions (e = 32.8 cm^2^/μmol) and 0.5, the moles of quinoneimine dye produced with 1 mol of H_2_O_2_.

### 2.3. Enzyme Linked Immunosorbent Assays

Levels of beta trace protein (Lipocalin-type prostaglandin D synthase) in hematoma fluid were quantified using the lipocalin-type, Human Prostaglandin D Synthase (Catalogue number: 10007684, Cayman Chemical, Ann Arbor, MI, USA) kits, according to the instructions provided by the manufacturers. A 1:100 dilution was used for the determination of beta trace concentration in hematoma.

### 2.4. Statistical Analysis

Data exhibiting normal distributions (Shapiro–Wilk tests) are presented as mean and standard deviation (SD), otherwise, as median and interquartile range (IQR). The differences between groups for continuous variables were calculated using the independent-samples *t*-test for data following normal distribution or the Mann–Whitney U test for data not following normal distribution. Correlations were assessed using the Spearman rank-order correlation coefficient. Categorical data are presented as frequencies and percentages. A two-sided *p* value of <0.05 was considered statistically significant. All statistical tests were performed using SPSS, version 25.0 (IBM Corp., Armonk, NY, USA).

## 3. Results

### 3.1. Patient Demographics, Medical History and Clinical Findings

Table 1 shows the demographic profile, medical history and clinical findings of the enrolled CSDH patients. There were twenty-eight CSDH patients (57.1% male) with a median age (IQR) of 80.5 years (72–86). Trauma to the head (e.g., due to a fall) could be remembered by patients or their relatives in the majority of cases (60.7%). Most cases displayed unilateral hematomas on CT (71.4%). Anticoagulant or antiplatelet use was evident in 46.4%. Hemiparesis was the most common symptom at admission.

### 3.2. Autotaxin Activity and Beta Trace Levels

**Figure 1:**
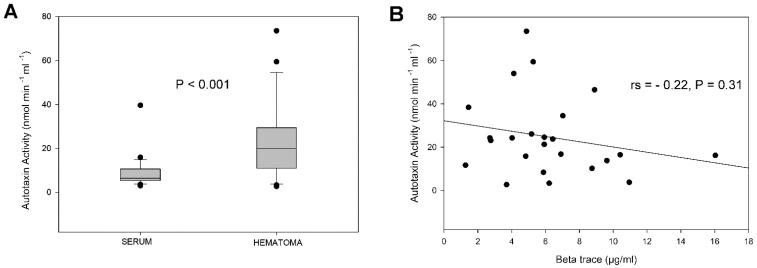
(**A**). Boxplot of the median (vertical lines inside boxes), 10th, 25th, 75th and 90th percentiles and outlying values (shown as dots) for autotaxin (ATX) activity in serum and hematoma fluid. There was a highly significant difference in ATX activity levels between serum and hematoma (P < 0.001) (**B**). Scatter plot of ATX activity and beta trace protein levels. The two variables did not show a significant correlation.

ATX activity was quantified in all patients. Hematoma fluid ATX activity [median (IQR) = 20 nmol min^−1^ mL^−1^ (11–29.5)] was significantly higher (P < 0.001, Figure 1A) than serum [median (IQR) = 6.5 (5.4–10.7) nmol min^−1^ mL^−1^]. Moreover, a sex-related difference in the activity of ATX in serum, but not in hematoma, was found. Thus, female patients displayed significantly higher (P = 0.026) serum ATX activity compared to male patients [median (IQR) = 9.0 (5.9–13.7) vs. 5.6 (3.9–7.1)]. There were no significant differences in serum or hematoma ATX activity between patients with a history of antiplatelet or anticoagulant medication and those without, between patients with an established history of head trauma prior to CSDH and those with no such history or between patients with a homogenous hematoma and those with a non-homogenous hematoma. ATX activity in serum and hematoma did not display a significant correlation. There was no significant correlation between age and ATX activity in either serum or hematoma.

The beta trace was quantified in a sub-cohort of 24 patients (9 females and 15 males). It was detected in all 24 hematoma fluid samples but did not display any significant correlation with ATX activity (Figure 1B). There were no significant differences in hematoma beta trace levels between sexes, between patients with a history of antiplatelet or anticoagulant medication and those without, between patients with a history of head trauma prior to CSDH and those with no such history or between patients with a homogenous hematoma and those with a non-homogenous hematoma. There was no significant correlation between age and beta trace in hematoma fluid.

## 4. Discussion

To the best of our knowledge, this is the first study investigating ATX activity in CSDH patients. From an analytical perspective, ATX activity is considered a valid indicator of ATX protein and LPA levels [24]. Moreover, an additional advantage of ATX analysis in biofluids over the analysis of LPA is its stability. In this regard, LPA has been shown to display variation with changes in blood processing/handling, temperature and storage time [25]. The present results of higher serum ATX activity in females compared to males are consistent with sexual dimorphism indicated in several previous clinical studies for both circulating ATX activity [26] and ATX protein levels [19,27], including protein levels in older patient cohorts [28]. The present findings did not indicate a significant difference in ATX activity levels between hematoma with a homogenous vs. non-homogenous [23] appearance in CT. Nevertheless, future analysis of the ATX-LPA axis in relation to CSDH and architecture as well as density should incorporate larger patient cohorts, allowing for a more detailed analysis of CSDH imaging sub-types [29].

One of the hypotheses tested in the present study was that CSF is a source of ATX in CSDH. In line with this hypothesis are previous clinical findings indicating abundant ATX synthesis in the human choroid plexus [14], high levels of ATX activity in CSF [16] and the presence of the CSF marker beta trace in the vast majority of hematomas [12]. Furthermore, additional clinical studies on CSDH pathogenesis show that a proportion of CSDH progresses from a subdural CSF collection [13]. While the present findings showed higher ATX activity in hematoma compared to serum, a significant correlation between ATX activity and BT in hematoma could not be established. A lack of correlation does not preclude CSF as one of the potential sources of ATX in CSDH. In this regard, CSF entry into the subdural cavity has been previously suggested to subside/cease following the completion of the hematoma membrane formation [30]. Moreover, in addition to CSF and blood-derived ATX, another potential ATX source is local synthesis from hematoma membrane cells such as fibroblasts, endothelial cells or macrophages, previously shown to express the enzyme at different sites under inflammatory conditions in human [31] and/or animal paradigms [32,33].

Chronically enhanced ATX-LPA axis activity may contribute to the propagation of several pathophysiological processes in CSDH. While LPA represents an established stimulus for human platelet aggregation and tissue factor expression, the major initiator of blood coagulation [6], it has also been shown to increase soluble human thrombomodulin, an anticoagulant, during vessel injury and endothelial cell damage [34]. It is, thus, noteworthy that levels of thrombomodulin are significantly higher in hematoma fluid of CSDH compared to peripheral blood, a finding that has been associated with hematoma outer membrane vessel damage, inhibition of hematoma thrombus formation and hematoma expansion [35]. Moreover, the actions of LPA on endothelial cells can lead to changes in vascular permeability [36] or sprouting angiogenesis [9].

Other than endothelial cells, enhanced ATX-LPA axis activity may influence numerous additional cellular targets, including smooth muscle cells, macrophages and lymphocytes, sustaining inflammatory cytokine/chemokine production and immune cell recruitment in CSDH [37]. Conversely, given evidence for enhanced local levels of inflammatory cytokines, such as IL-6 in CSDH [38], of note, is that IL-6 has been shown to stimulate ATX synthesis, thus, generating positive feedback amplification loops [31].

The ATX activity in the hematoma fluid of CSDH, shown for the first time in the present study, implicates the ATX-LPA axis in CSDH pathophysiology. It nevertheless generates numerous questions. First and foremost, the extent to which ATX protein and LPA in CSDH show the local increase that is predicted with the present findings. In this regard, experiments are underway to assess protein levels and the profile of molecular species of LPA in CSDH [33], taking into consideration the challenges associated with LPA analysis [25,39]. Moreover, additional research is warranted to establish the association of ATX-LPA axis components, including LPA receptors, with processes characterized by CSDH pathophysiology, such as local hypercoagulation/hyperfibrinolysis cycles, inflammation and angiogenesis [11]. Further, the utility of the ATX-LPA axis components as biomarkers of CSDH, architecture/density and recurrence remains to be established. Investigating several of these considerations will require analysis of patient cohorts significantly larger than in the current study, a parameter that represents a current limitation. Finally, ATX inhibitors are gaining traction in clinical trials against chronic inflammatory and fibrotic diseases [40,41]. Their utility in alleviating analogous processes that have been intimately associated with CSDH pathogenesis, development and recurrence warrants further investigation.

## Figures and Tables

**Table 1:** Demographic profile, medical history and clinical findings of chronic subdural hematoma patients.

Demographics and Medical History	Value
**Age** [median (IQR)] years	80.5 (72–86)
**Sex, N (%)**	
Male	16 (57.1)
Female	12 (42.9)
**Trauma, N (%)**	
Remembered/Established	17 (60.7)
Not Established	11 (39.3)
**CT findings, N (%)**	
Unilateral	20 (71.4)
Bilateral *	8 (28.6)
Homogenous	8 (8.6)
Non-homogenous **	20 (71.4)
**Neurological Deficits, N (%)**	
Hemiparesis	16 (57.1)
Headache	7 (25)
Disorientation	4 (14.3)
Seizure	1 (3.6)
Aphasia	6 (21.4)
Dysarthria	2 (7.1)
**Antiplatelet or**	
**Anticoagulant**	
YES	13 (46.4)
NO	15 (53.6)

* One sided sampling for bilateral hematomas. ** Non-homogenous hematomas were all cases falling into one of the three categories of non-homogenous categories defined by [23].

## Data Availability

Not applicable.
